# Retraction: Ethnobotanical inventory and medicinal perspectives of herbal flora of Shiwalik mountainous range of District Bhimber, Azad Jammu and Kashmir, Pakistan

**DOI:** 10.1371/journal.pone.0286782

**Published:** 2023-06-14

**Authors:** The PLOS ONE Editors

The *PLOS ONE* Editors retract this article [1] because it was identified as one of a series of submissions for which we have concerns about authorship, competing interests, and peer review. We regret that the issues were not addressed prior to the article’s publication.

MI, IH, TH, and MA did not agree with the retraction. HK, AP, MM, ST, FS, and HOE either did not respond directly or could not be reached.

## References

[1] Ishtiaq M, Khanum H, Hussain I, Parveen A, Maqbool M, Thind S, et al. (2022) Ethnobotanical inventory and medicinal perspectives of herbal flora of Shiwalik mountainous range of District Bhimber, Azad Jammu and Kashmir, Pakistan. PLoS ONE 17(3): e0265028. 10.1371/journal.pone.0265028 35349579 PMC8963552

